# Acute cerebral infarction with adenomyosis in a patient with fever: a case report

**DOI:** 10.1186/s12883-020-01787-0

**Published:** 2020-05-25

**Authors:** Yuan Zhao, Yongbo Zhang, Yishu Yang

**Affiliations:** Department of Neurology, Beijing Friendship Hospital, Capital Medical University, Beijing, 100050 China

**Keywords:** Adenomyosis, Acute cerebral infarction, Fever, Infection, Menstruation, CA125

## Abstract

**Background:**

It is reported that acute cerebral infarction with adenomyosis is associated with elevated D-Dimer, elevated CA125, anemia and menstruation. However, previous reports did not notice infection known as fever, which may be a potential risk factor for developing acute cerebral infarction with adenomyosis.

**Case presentation:**

We describe a 34-year-old woman who presented headache and fever (38 °C) for 4 days and left limb weakness for 1 day during her menstrual phase. Laboratory test data showed: Hemoglobin (HGB) (112 g/L, normal: 120–150 g/L), Carcinoembryonic antigen 125 (CA125) (937.70 U/ml, normal: 0–35 U/ml), D-Dimer (27.4 mg/L, normal: 0–1.5 mg/L). Magnetic resonance imaging (MRI) indicated acute cerebral infarction in right basal ganglia and subcortical region of right frontotemporal lobe. Further, brain computed tomography angiography (CTA) showed that the M1 segment of right middle cerebral artery was strictured and the distal branches of right middle cerebral artery were significantly less than those on the opposite side. No obvious abnormality was found in cranial magnetic resonance venogram (MRV). She had a 5-year history of adenomyosis. No tumors were found by whole body positron emission tomography-computed tomography (PET-CT). We treated this patient by using anti-infective therapy for 1 week and using anticoagulant therapy with low molecular weight heparin for 2 weeks. Subsequently, the anticoagulant therapy was discontinued and replaced by antiplatelet therapy with clopidogrel. We followed up this patient for 4 months, and no recurrence of cerebral infarction was found.

**Conclusions:**

Acute cerebral infarction with adenomyosis may be related to elevated D-Dimer, elevated CA125, anemia and menstruation. Our report suggests that infection may be a potential risk factor for developing acute cerebral infarction with adenomyosis.

## Background

Adenomyosis is a benign uterine disease. Histopathologically, adenomyosis is characterized by the presence of ectopic endometrial tissue (endometrial glands and/or stroma) in the myometrium, surrounded by proliferation and hypertrophic smooth muscle [1]. Adenomyosis mostly occurs in women of childbearing age and mainly manifested as dysmenorrhea, menorrhea and infertility. Acute cerebral infarction with adenomyosis in a patient with fever has been rarely reported.

Previous reported cases of acute cerebral infarction with adenomyosis were focused on middle-aged women over 35 years old. Here, we report a case of cerebral infarction in a 34-year-old young woman with adenomyosis. Besides, previous reports pay less attention to fever of patients with adenomyosis. In our report, acute cerebral infarction with adenomyosis was accompanied by fever, anemia, menstruation, elevated levels of CA125 and D-Dimer. Fever is one of the symptoms of infection. We discussed the factors associated with acute cerebral infarction with adenomyosis.

## Case presentation

A 34-year-old female patient presented headache and fever for 4 days and left limb weakness for 1 day was admitted to the hospital. Four days before admission, the patient had fever during menstruation, temperature up to 38 °C, paroxysmal headache, lower abdominal pain, muscle soreness, intermittent cough, sputum. Further, she did not present dizziness, nausea and vomiting. One day before admission, the patient had left limb weakness, left mouth angle askew and vague speech. The patient was treated in emergency department of our hospital. She had a history of adenomyosis for 5 years, which was treated with triprillin acetate, estradiol valerate, dydrogesterone and aspirin. However, 6 months before admission, the above-mentioned drugs have been stopped due to the poor treatment effect for adenomyosis in this patient. Bilateral thyroidectomy for thyroid cancer was performed 2 years before admission. At present, she takes 2 tablets of euthyrox orally every day. The patient denied hypertension, diabetes, hyperlipidemia, coronary heart disease and family history of cerebrovascular disease. She has no history of smoking. The highest body temperature was 39.1 °C after admission. Physical examination of nervous system indicated sleepiness, vague speech, left central facial-lingual paralysis, other cranial nerve examinations being normal, left limb muscle strength IV, right limb muscle strength V, limb muscle tension being normal, limb tendon reflex symmetry. Bilateral needling sensations were normal. Left Babinski’s and Pussep’s signs were positive. Meningeal irritation sign was negative. NIHSS score was 3 points. Blood pressure was 120/70 mmHg. Pulse was 80 times per minute and oxygen saturation was 97%. During auscultation, the breathing sounds of both lungs were thick, and wet rales were heard in bilateral lower lung. Chest X-ray showed suspicious bilateral lower pneumonia. The patient was diagnosed with pulmonary infection after admission.

**Fig. 1:**
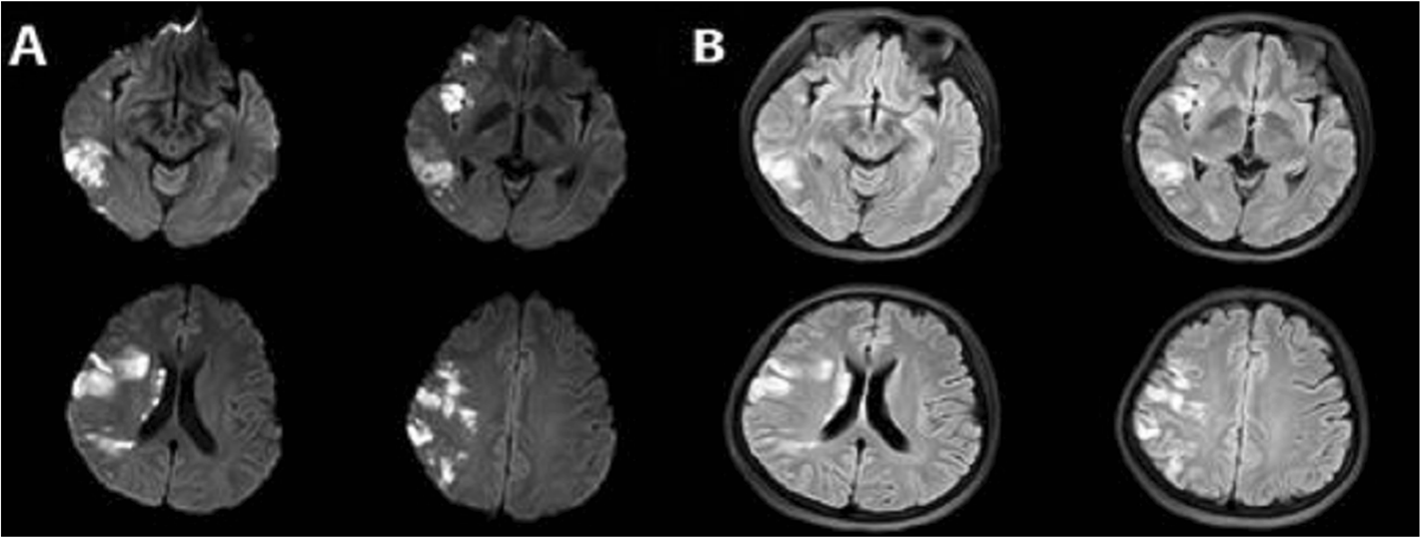
The MRI after two days of onset. Acute cerebral infarction in subcortical of right frontotemporal lobe and basal ganglia. **a** Diffusion weighted imaging (DWI). **b** Fluid-attenuated inversion recovery (FLAIR)

**Fig. 2:**
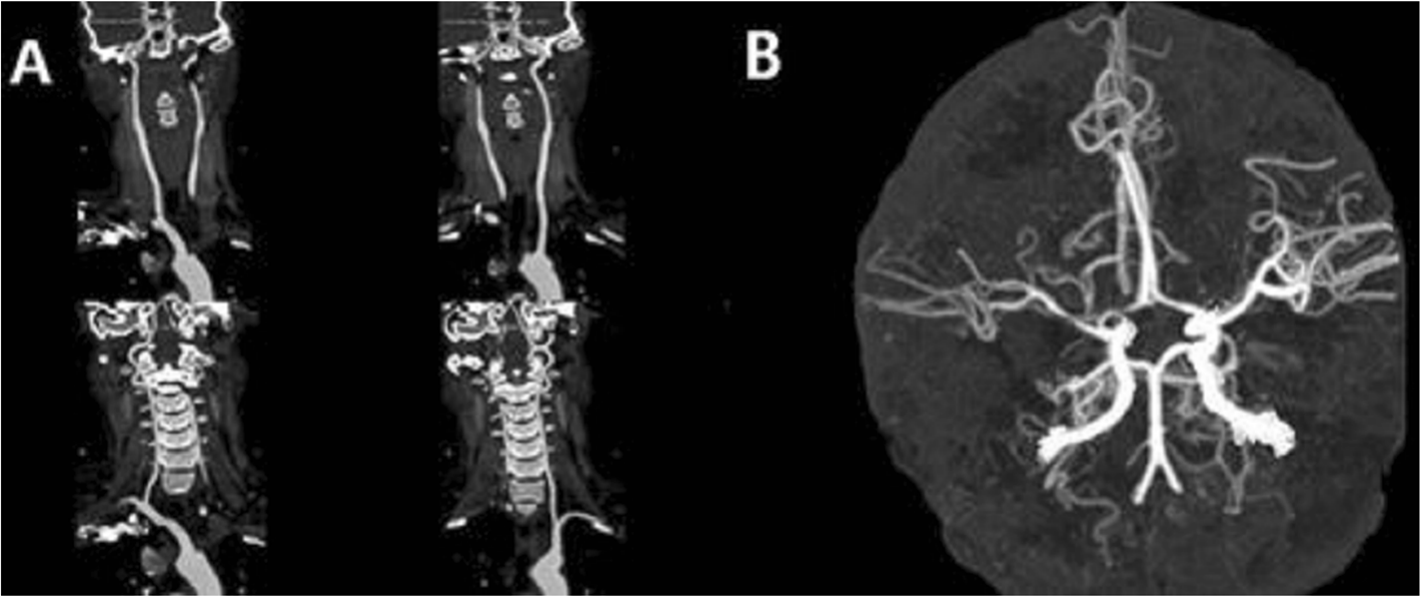
Brain CTA after six days of onset. **a** No abnormal carotid artery was found. **b** Stenosis was found in the M1 segment of the right middle cerebral artery and the distal branches were significantly less than those of the opposite side

**Fig. 3:**
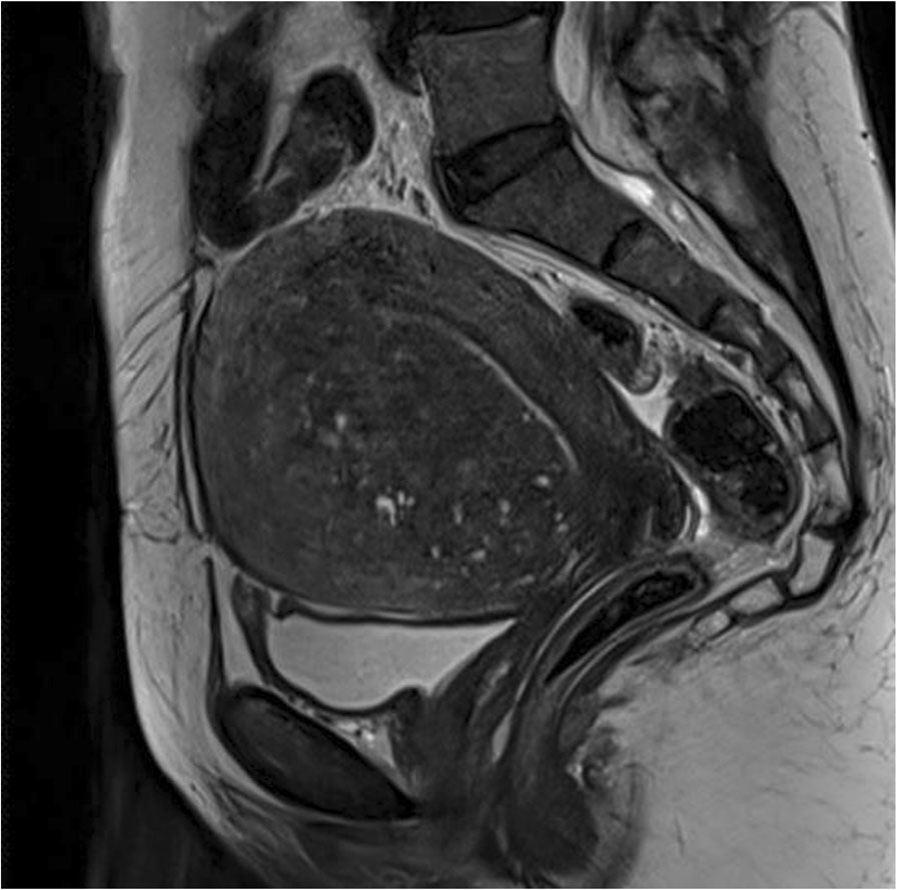
T2-weighted pelvic MRI revealed enlargement of the uterus, diffusely thickened junctional zone, the junctional zone and the muscle demarcation line blurring, suggesting adenomyosis

Blood routine indicated the total number of white blood cells (12.06 × 10^9^/L, normal: 3.5–9.5 × 10^9^/L), Neutrophil percentage (84.2%, normal: 40–75%), HGB (112 g/L, normal: 120–150 g/L). Results of admission examination showed elevated CRP (149 mg/L, normal: 0–8 mg/L), D-Dimer (27.4 mg/L, normal: 0–1.5 mg/L), fibrin (−ogen) degradation products (FDP) (69.20 mg/L, normal: 0–5 mg/L), fibrinogen (Fbg) (4.79 g/L, normal: 1.7–4 g/L), CA125 (937.7 U/ml, normal: 0–35 U/ml), NSE (39.51 ng/ml, normal: 0–18 ng/ml), CYFRA211 (3.65 ng/ml, normal: 0–3.3 ng/ml). Other tumor markers including AFP, CEA, CA199, CA153, CA724 and CA-50 were in normal range. Myocardial enzyme (CK, CK-MB, TNI, TNT) levels were in normal range. ESR, Anti chain “O”, Rheumatoid factor, ANCA, ANA, ENA, Immunoglobulin and complement were in normal range. Anticardiolipin antibody, Protein S and protein C were negative. Thyroid uptake (TU), T3, T4, FT3, FT4, thyroid stimulating hormone (TSH), anti-thyroglobulin antibodies (ATG-Ab), Antithyroperoxidase antibody (ATPO) were in normal range. Sparganum mansoni-IgG antibody, Filaria antibody, Lyme-IgG antibody, Cysticercosis-IgG antibody, Leishmania spp-IgG antibody and Guangzhou roundworm-IgG antibody in blood were negative. Result of rose bengal plate test was negative. Result of tuberculosis infection T cell detection was negative. HIV antibody, HCV antibody, HBsAg, HBeAg and Anti-HBc, Treponema pallidum antibody were negative. Cerebrospinal fluid analysis showed normal cerebrospinal fluid pressure, cell number, glucose and chloride of cerebrospinal fluid. Amphiphysin, CV2, PNMA2, Ri, Yo, Hu in cerebrospinal fluid and blood were negative. Magnetic resonance imaging (MRI) indicated acute cerebral infarction in right basal ganglia and subcortical region of right frontotemporal lobe (Fig. 1 a and b). Further, the computed tomography angiography (CTA) in the head showed stenosis in the M1 segment of right middle cerebral artery and the distal branches of right middle cerebral artery were significantly less than those on the opposite side (Fig. 2 a and b). The computed tomography angiography (CTA) in the neck showed no carotid stenosis or carotid dissection. No obvious abnormality was found in cranial magnetic resonance venogram (MRV). No tumors were found by whole body positron emission tomography-computed tomography (PET-CT). Transabdominal gynecological ultrasound suggested adenomyosis. Pelvic MRI (Fig. 3) revealed adenomyosis. Transthoracic echocardiography (TTE) did not indicate patent foramen ovale or valve disease. The examination of 24 h dynamic electrocardiogram (DCG) did not show paroxysmal atrial fibrillation or other arrhythmia. Ultrasound examination of veins of lower extremities did not present venous thrombosis.

We treated this patient by using anti-infective therapy (meropenem for injection) for 1 week and using anticoagulant therapy with low molecular weight heparin for 2 weeks. The anticoagulant therapy was discontinued and replaced by antiplatelet therapy with clopidogrel when D-Dimer was normal. CA125 was 202.5u/ml on the 20th day after onset. Transvaginal Gynecological Ultrasound suggested adenomyosis on the third month after onset. We followed up this patient for 4 months, and no recurrence of cerebral infarction was found.

## Discussion and conclusions

We reported a case of acute cerebral infarction with adenomyosis in a 34-year-old woman with fever. The patient had fever 4 days before admission and was diagnosed with pneumonia. The patient had acute cerebral infarction after she had pulmonary infection for 3 days. It has been reported that acute cerebral infarction with adenomyosis may be related to elevated D-Dimer, elevated CA125, anemia and menstruation. However, infection as a potential risk factor for developing acute cerebral infarction with adenomyosis is not reported yet.

Regarding the pathophysiologic mechansim of acute cerebral infarction with adenomyosis, we consulted previous cases and found 13 patients in the cases [2–8]. The median age of the 13 patients was 45.4 years. Most of the patients presented anemia, elevated levels of CA125 and D-Dimer (Table 1). 8 of the 13 patients had bilateral cerebral infarction, 1 of the 13 patients had unilateral stenosis of the right posterior cerebral artery, and 5 of the 13 patients had unilateral middle cerebral artery stenosis or occlusion. Embolus in heart was found in 2 patients (case 6, 8). 2 patients had embolism at other sites (finger in case 1, kidney in case 2). One case showed that adenomyosis can cause hypercoagulable state or diffuse intravascular coagulation (DIC), even lead to organ necrosis [9]. Besides, one case reported a patient with adenomyosis presented fever during menstrual phase, another case showed a patient with adenomyosis presented fever, but none of the two cases mentioned the effect of fever in a patient with acute cerebral infarction with adenomyosis [5, 6].

**Table 1:** summary of cases of acute cerebral infarction with adenomyosis

Case	Age range	CA125 (U/ml)	D-dimer (μg/ml)	HGB (g/l)	Fever	Menstr-ual phase	CI (Unilateral or bilateral)	Cerebrovascular involvement
1 [2]	45–50	159	1.1	84	–	no	bilateral	normal
2 [2]	40–45	–	–	70	–	–	Unilateral	normal
3 [2]	50–55	42.6	0.57^a^	69	–	yes	Unilateral	normal
4 [2]^b^	40–45	1750	6	86	–	yes	bilateral	normal
5 [3]^c^	55–60	334.8	7	–	–	–	bilateral	–
6 [4]^d^	45–50	379	3.99	99	no	no	bilateral	normal
7 [5]^b^	40–45	2115	17	103	yes (37.7 °C)	yes	bilateral	severe stenosis in MCA
8 [6]^d^	45–50	901	1.9	85	yes (37.4 °C)	–	bilateral	occlusion of the left M1 of MCA
9 [7]	40–45	395	1.4	–	–	–	Unilateral	occlusion of the left M2 of MCA
10 [7]	50–55	143	3.7	–	–	–	Unilateral	occlusion of the left M1 of MCA
11 [8]	30–35	937.1	1.050	134^a^	–	yes	bilateral	normal
12 [8]	35–40	735.7	2.34	108	–	yes	Unilateral	normal
13 [8]	45–50	546.5	12.04	121^a^	–	yes	bilateral	stenosis of the right PCA
14	34	937.7	27.4	112	yes	yes	Unilateral	severe stenosis in the right M1 of MCA

- indicates not mention; ^a^ indicates normal; ^b^ indicates the patient suffers from recurrence of acute cerebral infarction. ^c^ indicates the patient suffers from thrombus formation on the aortic valve. ^d^ indicates the patient suffers from nonbacterial thrombotic endocarditis

The reported factors in previous cases associated with acute cerebral infarction with adenomyosis are increased D-Dimer, elevated CA125, anemia and menstruation. One of the factors is increased D-Dimer. D-Dimer can increase if the amount of fibrin formation is excessive or fibrinolysis is rapid and excessive [9]. Further, D-Dimer can indicate activation of coagulation and fibrinolysis system, which may lead to thrombosis or menorrhea [10]. Another factor is elevated CA125. CA125 is a typical mucin molecule, which can activate the coagulation system by activating factor X. CA125 is considered as a susceptible factor for non-bacterial thrombotic endocarditis and disseminated thrombosis [11]. Besides, another factor is anemia. Anemia is considered as a hyperkinetic state which disturbs endothelial adhesion molecule genes that may lead to thrombus formation [12]. Moreover, disorder of coagulation system can also occur during menstruation [10, 13]. During non-menstrual period, a patient with adenomyosis has normal coagulation system. During menstrual period, FDP can be increased, APTT and PT can be prolonged. Therefore, menstruation may be related to the hypercoagulable state and may be one of the inducing factors of acute cerebral infarction.

However, previous reports did not pay attention to the effect of fever in a patient with acute cerebral infarction with adenomyosis. Here, we report a patient who had fever 4 days before admission and was diagnosed with pulmonary infection. Fever is one of the symptoms of pulmonary infection. One day before admission, the patient had left limb weakness. We treated this patient by using anti-infective therapy (meropenem for injection) for 1 week. The temperature of the patient was normal at third day after anti-infective therapy.

It is reported that infection can increase the incidence of acute cerebral infarction [14–17]. The cause of acute cerebral infarction with infection relates to infection load, changes in lipid metabolism, increased plasma fibrinogen, platelet activation or aggregation, platelet lysis, hypercoagulability, endothelial dysfunction, vascular smooth muscle spasm, unstable atherosclerosis and subsequent plaque rupture [14]. The association between acute infection and stroke is not dependent on particular microbial agents, but rather results from the inflammatory response to infection, which induces a procoagulant state [16]. Another research suggested that infection-induced immune responses may affect human proteins associated with stroke and cross-reactivity as a potential mechanistic link between infections and stroke [17]. Thus, infection and acute cerebral infarction may be linked by the procoagulant state induced by inflammation.

Besides, a case showed that increased CA125 and D-dimer in a patient with adenomyosis were detected during menstruation, indicating activated coagulation system associated with CA125 [8]. Further, another case indicated that patients with adenomyosis are at risk of having an activated coagulation system, which leads to increased risk of thrombotic disorders [9]. Thus, adenomyosis may be associated with activated coagulation system.

In our report, acute cerebral infarction with adenomyosis was accompanied by pulmonary infection. Our patient had acute cerebral infarction with adenomyosis after she had pulmonary infection for 3 days. We think that infection may play a role in the abnormal coagulation of a patient with acute cerebral infarction with adenomyosis. Further, we suggest that infection is a potential risk factor for developing acute cerebral infarction with adenomyosis.

Regarding the treatment for acute cerebral infarction with adenomyosis, it was reported that antiplatelet or anticoagulation combined gonadotropin-releasing hormone (GnRH) agonist was used in the treatment of acute cerebral infarction with adenomyosis. However, a report showed that warfarin and new oral anticoagulant (NOAC) were ineffective in the treatment of cerebral infarction with adenomyosis, as the patient in the report had cerebral infarction again after having new oral anticoagulant. This report suggested that adenomyosis resection may be the most effective therapy for preventing cerebral infarction [2]. Although it was reported that benign tumor resection can improve the coagulation function of patients, reports about benign tumor resection are few [2, 18]. At present, the efficacy of surgical hysterectomy in the treatment of acute cerebral infarction with adenomyosis is rarely reported.

We treated this patient by using anti-infective therapy (meropenem for injection) for 1 week and using anticoagulant therapy with low molecular weight heparin for 2 weeks. Subsequently, the anticoagulant therapy was discontinued when D-Dimer was normal and replaced by antiplatelet therapy with clopidogrel. Our treatment was based on the factors associated with activated coagulation system, which may include elevated D-Dimer, elevated CA125, anemia and infection during menstrual phase. We suggest it needs further research in clinical practices whether a patient with adenomyosis having elevated D-Dimer, elevated CA125, anemia and infection during menstrual phase can use anti-platelet and anticoagulant drugs in advance, so as to avoid acute cerebral infarction.

The brain CTA of our 34-year-old patient suggested that M1 segment of the right middle cerebral artery was obviously narrowed. We think that the narrowed M1 segment may be related to thrombosis due to changes of coagulation function caused by adenomyosis.

The CA125 level of the patient was high on admission. Six months before admission, the adenomyosis treatment including dydrogesterone has been stopped due to the poor treatment effect for adenomyosis in this patient. Therapy with dydrogesterone can suppress the ovarian function and promote atrophy of endometrium. Since CA125 is expressed in the endometrium, CA125 level may decline with atrophy of endometrium. Thus, high CA125 level on admission in the patient might be associated with cessation of adenomyosis treatment.

The CA125 level decreased in the 20th day after onset in our patient. Since CA125 is expressed in the peritoneum, pleura, pericardial membrane,and endometrium, high level of CA125 can also be seen in pregnancy, menstruation and endometriosis [6]. The elevation of serum CA125 during menstruation is thought to be related with endometrial cell surface antigen shed into the systemic circulation or peritoneal irritation [8]. Thus, the CA125 level may be associated with the menstrual cycle.

In summary, we discussed the factors associated with acute cerebral infarction with adenomyosis, which may include increased D-Dimer, elevated CA125, anemia and menstruation. Besides, we think that infection may also play a role in a patient with acute cerebral infarction with adenomyosis. Further, we suggest that infection is a potential risk factor for developing acute cerebral infarction with adenomyosis and should be paid attention to at the time of diagnosis for a patient with adenomyosis.

Since our study reported acute cerebral infarction with adenomyosis in one patient with infection, more cases of acute cerebral infarction with adenomyosis accompanied with infection need to be noticed and researched.

## Data Availability

All data related to this case report are contained within the manuscript.

## Abbreviations

HGB: Hemoglobin
CA125: Carcinoembryonic antigen
MRI: magnetic resonance imaging
CTA: computed tomography angiography
MRV: amagnetic resonance venogram
PET-CT: positron emission tomography-computed tomography
CRP: C-reactive protein
FDP: fibrin(−ogen) degradation products
Fbg: Fibrinogen
CK: Creatine kinase
CK-MB: Creatine kinase-muscle/brain
TNT: Troponin
TNI: Troponin I
ESR: Erythrocyte sedimentation rate
AFP: Alpha fetoprotein
CEA: Carcinoembryonic antigen
CA153: Cancer antigen 153
CYF211: Cytokeratin fragment 211
CA724: Cancer antigen 724
CA-50: Cancer antigen 50
TU: Thyroid uptake
TSH: Thyroid stimulating hormone
ATPO: Antithyroperoxidase antibody
ATG-Ab: anti-thyroglobulin antibodies
DWI: Diffusion weighted imaging
HCV: Hepatitis C virus
HIV: Human Immunodeficiency Virus
HBsAg: Hepatitis B surface antigen
HBeAg: Hepatitis e antigen
Anti-HBc: Anti-hepatitis B core antibody
DIC: diffuse intravascular coagulation
